# Geographic priorities in the global action plan for wasting: Burkina Faso, Ethiopia, Kenya and Malawi

**DOI:** 10.2471/BLT.25.293259

**Published:** 2026-03-30

**Authors:** Adriana Newman, Indi Trehan, Tessema Masresha, Kenneth M Maleta, Hama A Diallo, Moses Ngari, Daniel Muhinja, Kirkby D Tickell

**Affiliations:** a Department of Global Health, University of Washington, Seattle, WA, United States of America (USA).; b Department of Pediatrics, University of Washington, Seattle, USA.; c Nutrition, Environmental Health and Non-communicable Diseases Research Directorate, Ethiopian Public Health Institute, Addis Ababa, Ethiopia.; d Department of Nutrition and Dietetics, Kamuzu University of Health Sciences, Blantyre, Malawi.; e Department of Public Health, University Joseph Ki-Zerbo, Ouagadougou, Burkina Faso.; f KEMRI–Wellcome Trust Research Programme, Kilifi, Kenya.; g Save the Children, Kenya and Madagascar Country Office, Nairobi, Kenya.

## Abstract

Shifts in global aid funding threaten the sustainability of programmes addressing childhood wasting and population-based surveys used to inform their geographic targeting. Re-evaluating how priority regions are identified is essential to ensure constrained resources reach the greatest number of affected children. We examined how the *Global action plan on child wasting* prioritization strategies aligned with the geographic distribution of childhood wasting in four countries: Burkina Faso, Ethiopia, Kenya and Malawi. We used the two most recent Demographic and Health Surveys of each country to estimate the annual absolute burden of wasting in global action plan priority and non-priority areas. Prioritized areas consistently captured the highest wasting prevalence and, except in Kenya, experienced larger reductions in wasting prevalence over time. However, in Burkina Faso, Kenya and Malawi, between 43.6% (142 631/327 349) and 69.8% (992 370/1 422 301) of children with wasting lived in non-priority areas, largely in more populous regions with a lower prevalence of wasting but a high absolute burden. These findings highlight a key limitation of prevalence-based prioritization: low-prevalence areas can contain numerous affected children if they are more densely populated. While prevalence-based targeting has successfully delivered nutritional services to millions of children, targets of the global action plan and sustainable development goals are unlikely to be met without addressing the burden of wasting in non-priority areas. With global nutrition financing increasingly constrained, countries must adapt data systems to enable flexible prioritization that balances prevalence and burden, while implementing context-adapted strategies to cost-effectively expand coverage where the largest numbers of affected children live.

## Résumé

Les changements dans le financement mondial de l’aide menacent la pérennité des programmes de lutte contre l’émaciation infantile et des enquêtes démographiques utilisées pour définir leur ciblage géographique. Il est essentiel de réévaluer l’identification des régions prioritaires afin de garantir une livraison de ressources limitées pour le plus grand nombre d’enfants touchés. Nous avons examiné la correspondance entre les stratégies de priorisation du Plan d’action mondial contre l’émaciation infantile et la répartition géographique de cette affection dans quatre pays: le Burkina Faso, l’Éthiopie, le Kenya et le Malawi. Nous avons utilisé les deux dernières enquêtes démographiques et sanitaires de chaque pays pour évaluer la charge annuelle absolue de l’émaciation dans les zones prioritaires et non prioritaires du Plan d’action mondial. Les zones prioritaires présentaient systématiquement la prévalence d’émaciation la plus élevée et, à l’exception du Kenya, ont connu des réductions plus importantes de la prévalence d’émaciation au fil du temps. Toutefois, au Burkina Faso, au Kenya et au Malawi, entre 43,6% (142 631 sur 327 349) et 69,8% (992 370 sur 1 422 301) des enfants souffrant d’émaciation vivaient dans des zones non prioritaires, principalement des régions plus peuplées présentant une prévalence plus faible d’émaciation mais un fardeau absolu élevé. Ces résultats mettent en évidence une limite majeure de la hiérarchisation fondée sur la prévalence: les zones à faible prévalence peuvent compter de nombreux enfants touchés si elles sont plus densément peuplées. Si le ciblage fondé sur la prévalence a permis d’assurer les besoins nutritionnels de millions d’enfants, il y a peu de chances d’atteindre les objectifs de développement durable et ceux du Plan d’action mondial sans s’attaquer au fardeau de l’émaciation dans les zones non prioritaires. Face aux contraintes croissantes du financement mondial de la nutrition, les pays doivent adapter leurs systèmes de données pour permettre une hiérarchisation flexible qui concilie prévalence et fardeau, tout en mettant en œuvre des stratégies adaptées au contexte afin d’étendre de manière rentable la couverture dans les zones où vivent le plus grand nombre d’enfants touchés.

## Resumen

Los cambios en la financiación mundial de la ayuda amenazan la sostenibilidad de los programas dirigidos a la emaciación infantil y de las encuestas poblacionales utilizadas para orientar su focalización geográfica. Resulta esencial reevaluar la forma en que se identifican las regiones prioritarias para asegurar que los recursos limitados lleguen al mayor número posible de niños y niñas afectados. Se examinó cómo las estrategias de priorización del Plan de acción mundial sobre la emaciación infantil se alinean con la distribución geográfica de la emaciación infantil en cuatro países: Burkina Faso, Etiopía, Kenya y Malawi. Se utilizaron las dos encuestas demográficas y de salud más recientes de cada país para estimar la carga absoluta anual de emaciación en las áreas prioritarias y no prioritarias del Plan de acción mundial. Las áreas priorizadas captaron de forma consistente las mayores prevalencias de emaciación y, salvo en Kenya, experimentaron reducciones más pronunciadas de la prevalencia de emaciación con el tiempo. Sin embargo, en Burkina Faso, Kenya y Malawi, entre el 43,6% (142 631/327 349) y el 69,8% (992 370/1 422 301) de los niños y niñas con emaciación residían en áreas no prioritarias, en gran medida en regiones más pobladas con menor prevalencia de emaciación pero con una carga absoluta elevada. Estos hallazgos destacan una limitación clave de la priorización basada en la prevalencia: las áreas con baja prevalencia pueden albergar un gran número de casos si presentan una mayor densidad poblacional. Si bien la focalización basada en la prevalencia ha permitido proporcionar servicios nutricionales a millones de niños y niñas, es poco probable que se alcancen los objetivos del Plan de acción mundial y de los objetivos de desarrollo sostenible sin abordar la carga de la emaciación en las áreas no prioritarias. En un contexto de financiación mundial de la nutrición cada vez más restringida, los países deben adaptar sus sistemas de datos para permitir una priorización flexible que equilibre la prevalencia y la carga, al tiempo que se implementan estrategias adaptadas al contexto para ampliar la cobertura de manera rentable allí donde reside el mayor número de niños y niñas afectados.

## ملخص

إن التحولات الحادثة في تمويل المساعدات العالمية تُهدد استدامة البرامج التي تُعنى بمكافحة الهزال لدى الأطفال، والمسوح السكانية المستخدمة لتحديد استهدافها الجغرافي. تُعد إعادة تقييم كيفية تحديد المناطق ذات الأولوية أمرًا بالغ الأهمية لضمان وصول الموارد المحدودة إلى أكبر عدد ممكن من الأطفال المتضررين. قمنا بدراسة مدى التوافق بين استراتيجيات تحديد الأولويات في خطة العمل العالمية لمكافحة الهزال لدى الأطفال، والتوزيع الجغرافي لهزال الأطفال في أربع دول: بوركينا فاصو وإثيوبيا وكينيا وملاوي. لقد استخدمنا أحدث مسحين للسكان والصحة لكل دولة، وذلك لتقدير العبء السنوي المطلق للهزال في المناطق ذات الأولوية وغير ذات الأولوية في خطة العمل العالمية. وسجلت المناطق ذات الأولوية أعلى معدلات انتشار للهزال بشكل متسق، وشهدت، باستثناء كينيا، انخفاضات أكبر في معدلات انتشار الهزال بمرور الوقت. ومع ذلك، ففي بوركينا فاصو وكينيا وملاوي، تراوحت نسبة الأطفال المصابين بالهزال الذين يعيشون في مناطق غير ذات الأولوية بين %43.6 (142631/327349)، و%69.8 (992370/1422301)، وخاصة في المناطق الأكثر اكتظاظًا بالسكان مع معدل انتشار أقل للهزال، ولكن بعبء مطلق مرتفع. تسلط هذه النتائج الضوء على أحد أهم قيود تحديد الأولويات بناءً على معدل الانتشار: حيث يمكن أن تضم المناطق ذات معدل الانتشار المنخفض عددًا كبيرًا من الأطفال المتضررين إذا كانت ذات كثافة سكانية عالية. وبينما نجح الاستهداف القائم على معدل الانتشار في تقديم خدمات التغذية لملايين الأطفال، فمن غير المرجّح تحقيق أهداف خطة العمل العالمية وأهداف التنمية المستدامة دون معالجة عبء الهزال في المناطق غير ذات الأولوية. ومع تزايد القيود على التمويل العالمي للتغذية، يجب على الدول تكييف أنظمة البيانات لتمكين تحديد الأولويات بمرونة، بما يُوازن بين معدل الانتشار والعبء، مع تطبيق استراتيجيات متكيفة حسب الأوضاع لتوسيع نطاق التغطية بفعالية من حيث التكلفة في المناطق التي يعيش فيها أكبر عدد من الأطفال المتضررين.

## 摘要

全球援助资金转移可能会导致无法持续推进旨在解决儿童消瘦问题的项目，也无法持续开展基于人群的调查以提供确定优先关注地区所需信息。重新评估优先关注地区的确定方式是确保有限资源尽量惠及更多受影响儿童的关键所在。我们研究了《儿童消瘦问题全球行动计划》优先战略与埃塞俄比亚、布基纳法索、肯尼亚和马拉维这四个国家中儿童消瘦问题地理分布情况的契合程度。我们运用了以上各个国家的最近两次人口和健康调查相关数据，以估算《全球行动计划》所提及优先关注地区和非优先关注地区中因消瘦问题而造成的年度绝对负担。优先关注地区的消瘦患病率始终处于最高水平，但随着时间的推移，这些地区的消瘦患病率降幅也越来越大（肯尼亚除外）。然而，在布基纳法索、肯尼亚和马拉维，仍有 43.6% (142,631/327,349) 至 69.8% (992,370/1,422,301) 存在消瘦问题的儿童居住在非优先关注地区，且其中大多数居住在人口密度较大、消瘦患病率较低但绝对负担较重的区域。这些研究结果凸显了基于患病率的优先战略存在一个关键的局限性问题，即患病率较低的地区也可能会因为人口密度较大而存在大量受影响儿童。尽管基于患病率确定优先关注地区的战略已成功实施并为数百万儿童提供了营养服务，但是如果不能减轻非优先关注地区因消瘦问题而造成的负担，则《全球行动计划》目标以及可持续发展目标可能都无法得以实现。在全球营养资金愈发紧张的情况下，各国必须调整数据系统以在均衡考虑患病率和负担的前提下制定灵活优先战略，同时实施可根据实际情况进行相应调整的战略，以通过具有成本效益的方式将覆盖范围扩大至受影响儿童人数最多的地区。

## Резюме

Изменения в вопросах финансирования глобальных стратегий помощи ставят под угрозу стабильность работы программ по борьбе с истощением у детей, а также проведение популяционных исследований, необходимых для принятия решений о том, на какие регионы следует ориентировать такие программы. Пересмотр подходов к определению приоритетных регионов крайне важен для того, чтобы в условиях ограничения ресурсов охватить помощью как можно большее количество нуждающихся детей. Авторы изучили, насколько стратегии распределения приоритетов в Глобальном плане действий по борьбе с детским истощением соотносятся с географическим распределением истощения у детей в четырех странах: Буркина-Фасо, Кении, Малави и Эфиопии. Были использованы результаты двух последних исследований демографических характеристик и состояния здоровья для каждой из стран для оценки абсолютного годового бремени истощения в районах, которые Глобальный план действий определяет как приоритетные и неприоритетные. В приоритетных районах неизменно отмечалась наибольшая распространенность истощения, и, за исключением Кении, с течением времени в них наблюдалось более значительное сокращение распространенности истощения. Однако в Буркина-Фасо, Кении и Малави от 43,6% (142 631/327 349) до 69,8% (992 370/1 422 301) детей с истощением проживали в неприоритетных регионах, в основном более населенных с меньшей распространенностью истощения, но с более высоким абсолютным бременем. Эти результаты подчеркивают ключевое ограничение, связанное с определением приоритетности по распространенности истощения: в регионах с низкой распространенностью этого состояния может проживать значительное количество нуждающихся детей, если эти регионы более густо населены. Хотя целенаправленное распределение помощи на основании распространенности позволило успешно предоставить питание миллионам детей, достижение целей Глобального плана действий и реализация целей в области устойчивого развития представляются маловероятными без решения вопроса, связанного с бременем истощения в неприоритетных регионах. В условиях все более жестких ограничений глобального финансирования программ питания страны обязаны адаптировать системы данных, чтобы иметь возможность гибкой расстановки приоритетов, учитывающей баланс между распространенностью и бременем. Страны также должны одновременно внедрять адаптированные к местным условиям стратегии для экономически эффективного расширения охвата регионов, в которых проживает наибольшее количество нуждающихся детей.

## Introduction

Childhood wasting is one of the leading contributors to child mortality worldwide. In 2024, an estimated 42.8 million children were affected by wasting at any given time, including 12.2 million with severe wasting.^1^ Among children aged 6–59 months, wasting is diagnosed by a weight-for-height z-score (WHZ) < −2 or a mid-upper-arm circumference (MUAC) < 12.5 cm or the presence of bilateral pitting oedema. While interventions delivered through programmes within the Integrated Management of Acute Malnutrition have substantially reduced mortality,^2^ their effectiveness depends on sustained financing^3^ and the availability and appropriate use of reliable data to guide resource allocation. Recent funding disruptions and limitations in current prioritization models threaten these gains, necessitating urgent policy reassessment.

Cuts to international aid are undermining both the delivery of wasting interventions and the data systems that inform evidence-based prioritization. Many low- and middle-income countries have relied heavily on previous programmes funded by the United States Agency for International Development (USAID), such as the Demographic and Health Surveys (DHS), as a main source of nutritional and health data; for example, only 3% of surveys in 26 low-income and 8% in 41 lower-middle income countries are reported to be domestically funded.^4^ The 2025 USAID funding withdrawal has disrupted ongoing surveys, threatened the future of DHS programmes^5^ and placed programmes within the Integrated Management of Acute Malnutrition at risk. Without data infrastructure, countries may default to replicating previous policy decisions or relying on outdated data sets, which could result in inaccurate and inefficient allocation of increasingly limited resources.

Prevalence-based prioritization models may compound this issue. The *Global action plan on child wasting* provides a roadmap for the implementation of interventions under the Integrated Management of Acute Malnutrition by prioritizing subnational areas based on wasting prevalence, food insecurity, internal displacement, and sanitation and clean water access.^6^ Priority areas are often arid or semi-arid places with low population density, while non-priority areas are typically less arid but more densely populated. Non-priority areas with lower wasting prevalence, but higher population density, may have a higher absolute number of children with wasting than priority areas.

To inform the necessary policy adjustments to optimize interventions to treat wasting, we analysed how priority areas within the global action plan align with the geographic distribution of child wasting in four countries: Burkina Faso, Ethiopia, Kenya and Malawi.

## Prioritization and wasting burden

As part of the implementation of the global action plan, countries adopted national targets for treatment of wasting for 2025.^6^ Burkina Faso aimed to treat 87% of severe and 50% of moderate cases of wasting, while Kenya targeted 75% of severe and 50% of moderate cases annually. Malawi committed to treating 75% of total wasting cases yearly, whereas Ethiopia set absolute annual targets of treating 75 000 children with severe wasting and 1.5 million with moderate wasting.

To assess alignment between these national targets and the geographic distribution of child wasting, we estimated the absolute burden (number of affected children) of wasting at the subnational level and compared the burden with global action plan designations for these localities as priority and non-priority areas.^6^ In Kenya, non-priority counties were further stratified into non-arid and semi-arid counties to align with the classifications of the National Drought Management Authority.^7^ We estimated the annual subnational wasting burden in three steps. First, we obtained the subnational prevalence of wasting and population totals from each country’s two most recent DHSs,^8^^–^^15^ supplemented with national census data where subnational population totals were missing.^16^^–^^19^ Second, we derived the number of children younger than 5 years in each subnational territory by applying the national proportion of children younger than 5 years reported in the DHS to each subnational population total. Third, we estimated the annual burden of severe and total wasting by multiplying the population counts of children younger than 5 years by the prevalence of wasting adjusted using an incidence correction factor of 3.6 to account for incident cases arising over a year.^20^ We then aggregated these subnational estimates by global action plan designation to quantify the share of the national wasting burden within priority versus non-priority areas.

Because the DHS assesses nutritional status primarily using the WHZ score,^21^ these estimates of burden exclude children with kwashiorkor and children identified using MUAC. Given that children who are diagnosed as wasted by MUAC also have high mortality rates,^22^ their omission is a notable limitation.

Applying this analytic approach in the four countries revealed major differences in how geographic prioritization of the global action plan aligns with the national wasting burden.

### Burkina Faso

Burkina Faso illustrates the challenges of the current global action plan prioritization most clearly. The country prioritizes three of its 13 regions,^6^ all located in the northern Sahelian ecological belt, covering 19.7% (3 602 861/18 250 971) of the national population (online repository).^23^ These priority areas had the highest wasting prevalence among all the four countries and achieved the greatest progress, reducing the prevalence of severe wasting from 8.0% (48 747/608 895) in 2010 to 3.4%(18 963/554 841) in 2021.

However, priority regions accounted for only 33.0% (224 237/680 029) and 29.0% (87 230/300 321) of severe cases of wasting, and 31.3% (564 630/1 804 883) and 30.2% (429 931/1 422 301) of total cases of wasting in 2010 and 2021, respectively (Table 1). Highly populated non-priority regions such as Centre and Centre-Est consistently reported higher numbers of children with wasting than all but one priority region. Notably, the Centre-Est region has experienced political unrest and insecurity since 2016, which may contribute to its high wasting burden. This situation underscores the need for continued focus on childhood wasting beyond formally prioritized regions, particularly in humanitarian settings.

**Table 1:** Estimates of wasting in priority and non-priority areas of the Global Action Plan on Child Wasting, Burkina Faso, Ethiopia, Kenya and Malawi

Country	Severe wasting^a^				Total wasting^b^		
	Prevalence, %	Estimated burden, no. of children	Proportion of national burden, %		Prevalence, %	Estimated burden, no. of children	Proportion of national burden, %
**Burkina Faso **							
Priority (2010)	8.0 (48 747/608 895)	224 237	33.0		20.2 (122 746/608 895)	564 630	31.3
Non-priority (2010)	5.2 (99 085/1 914 212)	455 792	67.0		14.1 (269 620/1 914 212)	1 240 253	68.7
Priority (2021)	3.4 (18 963/554 841)	87 230	29.0		16.8 (93 463/554 841)	429 931	30.2
Non-priority (2021)	2.1 (46 324/2 255 809)	213 091	71.0		9.6 (215 733/2 255 809)	992 370	69.8
**Ethiopia **							
Priority (2011)	3.1 (333 706/10 671 882)	1 535 048	96.5		10.3 (1 102 736/10 671 882)	5 072 586	96.0
Non-priority (2011)	1.8 (11 944/670 861)	54 944	3.5		6.9 (46 322/670 861)	213 082	4.0
Priority (2019)	1.2 (108 993/8 800 838)	501 367	97.8		7.1 (627 632/8 800 838)	2 887 105	96.5
Non-priority (2019)	0.4 (2 470/553 243)	11 364	2.2		4.1 (22 655/553 243)	104 213	3.5
**Kenya^c^**							
Priority (2014)	2.2 (29 304/1 349 257)	105 494	47.5		9.4 (126 559/1 349 257)	582 170	51.2
Non-priority semi-arid (2014)	0.8 (9 775/1 283 322)	44 963	20.2		3.3 (42 335/1 283 322)	194 740	17.1
Non-priority non-arid (2014)	0.5 (15 605/2 928 446)	71 784	32.3		2.7 (78 466/2 928 446)	360 944	31.7
Priority (2022)	1.3 (20 081/1 513 247)	92 371	50.3		10.0 (150 758/1 513 247)	693 486	48.5
Non-priority semi-arid (2022)	0.6 (7 958/1 407 837)	36 607	19.9		5.1 (72 388/1 407 837)	332 986	23.3
Non-priority non-arid (2022)	0.4 (11 894/3 155 482)	54 713	29.8		2.8 (87 514/3 155 482)	402 565	28.2
**Malawi **							
Priority (2010)	2.0 (24 540/1 244 952)	112 885	70.4		4.5 (55 469/1 244 952)	255 158	59.3
Non-priority (2010)	1.0 (10 319/1 002 455)	47 466	29.6		3.8 (38 013/1 002 455)	174 860	40.7
Priority (2016)	0.7 (10 129/1 464 579)	46 595	72.8		2.7 (40 156/1 464 579)	184 718	56.4
Non-priority (2016)	0.3 (3 779/1 176 977)	17 381	27.2		2.6 (31 007/1 176 977)	142 631	43.6

^a^ Severe wasting corresponds to a weight-for-height z-score of < –3.

^b^ Total wasting corresponds to a weight-for-height z-score of < –2.

^c^ Kenyan non-priority regions are divided into semi-arid and non-arid because nutritional programme prioritization was extended to include non-priority semi-arid areas during droughts.

### Ethiopia

Ethiopia presents a contrasting approach. Six of its nine regional states were designated as priority areas,^6^ accounting for 94.1% (69 297 937/73 654 178) of the national population (online repository).^23^ Within these priority regions, the prevalence of severe wasting declined from 3.1% (333 706/10 671 882) in 2011 to 1.2% (108 993/8 800 838) in 2019. As a result, priority areas had 96.5% (1 535 048/1 589 992) and 97.8% (501 367/512 731) of severe wasting cases, and 96.0% (5 072 586/5 285 668) and 96.5% (2 887 105/2 991 318) of total wasting cases in the 2011 and 2019 surveys, respectively, while all non-priority regional states had fewer cases than even the lowest burden priority region (Table 1). This broad prioritization recognizes that wasting is a nationwide challenge, exacerbated by a series of climate, health and security crises and further intensified today by the lingering effects of the coronavirus disease 2019 (COVID-19) pandemic.

Of the four countries, Ethiopia is the only one whose prioritization makes the goals of the global action plan attainable. However, attempting to prioritize almost all of the country has made the delivery of interventions within the Integrated Management of Acute Malnutrition difficult to sustain. This difficulty is evidenced by low compliance rates, with only 19.4 (14/72) and 11.1 (8/72) of facilities meeting minimum standards for treating severe and moderate wasting cases, respectively.^24^

### Kenya

Kenya prioritizes 12 of its 47 counties,^6^ which represent 24.9% (11 730 595/47 105 164) of the national population, including northern arid and semi-arid counties, as well as large southern urban centres such as Nairobi (online repository).^23^ The inclusion of large cities acknowledges that wasting in urban areas is driven by factors beyond local ecology. Between 2014 and 2022, the prevalence of severe wasting in priority counties declined from 2.2% (29 304/1 349 257) to 1.3% (20 081/1 513 247), while reductions in non-priority counties were negligible (< 0.2%). Over the same period, the prevalence of total wasting increased nationwide, compounded by a major drought in 2022.

**Fig. 1:**
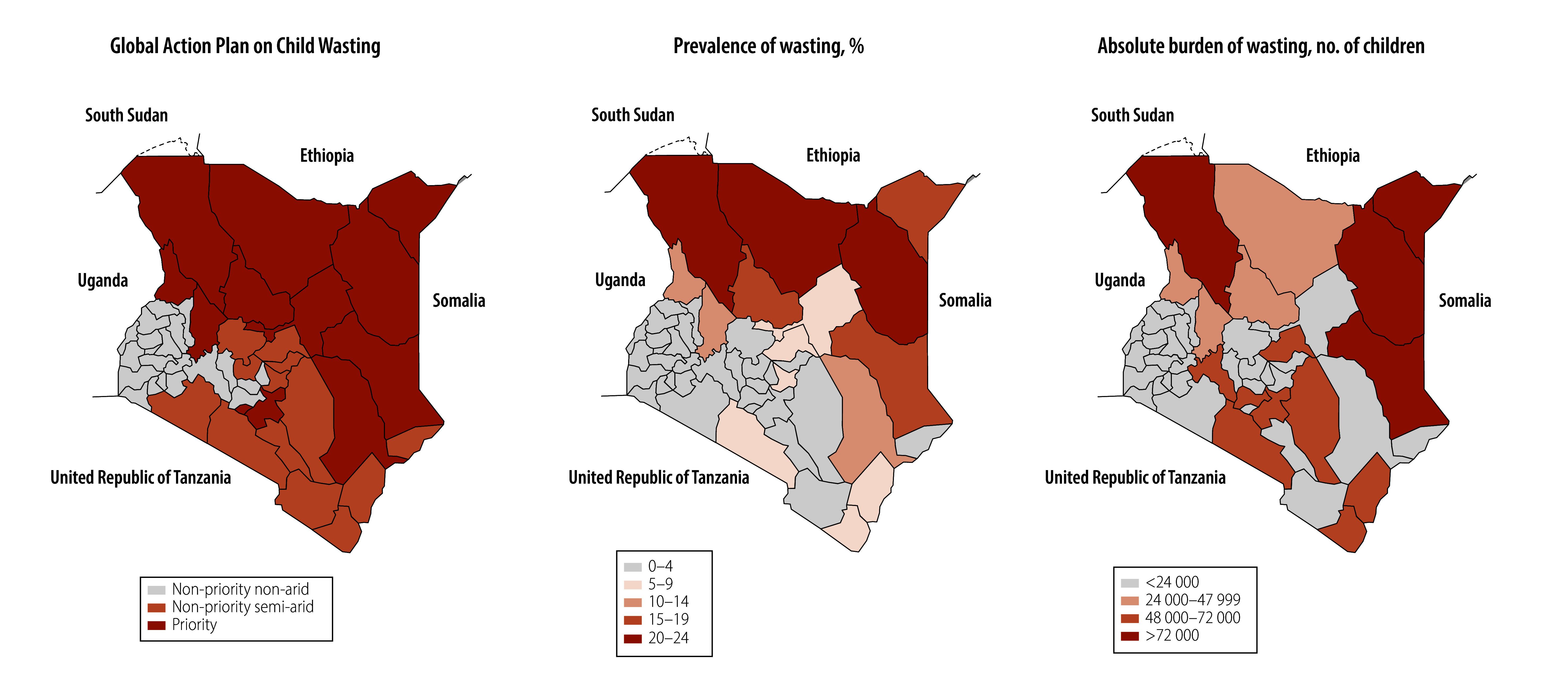
Distribution of wasting priority counties, prevalence and cases, Kenya, 2022

This prioritization strategy leaves a substantial share of the national burden of wasting outside designated priority areas with about half of all children with wasting living in non-priority counties: 48.8% (555 684/1 137 854) in 2014 and 51.5% (735 551/1 429 037) in 2022 (Fig. 1). Some non-priority counties (including Kiambu, Kilifi, Meru and Trans Nzoia) have a higher absolute burden of wasting than many priority counties. Non-arid, non-priority counties alone account for 29.8% (54 713/183 691) of severe wasting cases, leading to high rates of morbidity and mortality. For example, despite a low prevalence of wasting in Siaya (1.7%),^13^ wasting contributed to 44.1% of preventable child deaths.^25^ During droughts, emergency resources are extended to semi-arid counties outside the priority list; however, non-priority non-arid counties remain largely overlooked.

### Malawi

Malawi prioritizes 11 of its 28 districts,^6^ covering 55.4% (9 699 199/17 493 749) of the national population (online repository).^23^ Priority districts had a higher prevalence of severe wasting than non-priority districts but experienced the lowest decline in severe wasting among the four countries, from 2.0% (24 540/1 244 952) to 0.7% (10 129/1 464 579). Priority districts accounted for 70.4% (112 885/160 351) and 72.8% (46 595/63 976) of cases of severe wasting, and 59.3% (255 158/430 018) and 56.4% (184 718/327 349) of total wasting in 2010 and 2016, respectively. However, a sizeable proportion of the total wasting burden remains in non-priority districts. For example, Mulanje and Ntcheu districts had a greater proportion of children with severe or total wasting than some priority districts.

### Inferences of our findings

Taken together, these case studies show that the prevalence-based prioritization within the global action plan has successfully concentrated resources in areas of high nutritional risk. These areas consistently showed a higher prevalence of wasting and (except in Kenya) experienced larger reductions in prevalence between surveys. At the same time, in three of the four countries (Burkina Faso, Kenya and Malawi), between 43.6% (142 631/327 349) and 69.8% (992 370/1 422 301) of children with wasting live outside priority areas. These findings show a key limitation in prevalence-based prioritization: more densely populated regions with lower prevalence can contain large absolute numbers of affected children who remain underserved. As a result, sustainable development goal (SDG) 3.2.1,^26^ that is by 2030, end preventable deaths of newborns and children younger than 5 years, with all countries aiming to reduce neonatal mortality and under-5 mortality, and global action plan targets are unlikely to be achieved through prevalence-based prioritization alone.

## Recommendations

In the context of increasingly constrained funding, our findings demonstrate that achieving global action plan and SDG targets will require rethinking national wasting strategies. Health ministries need to find alternative data systems to inform decision-making, optimize geographic prioritization to reflect absolute burden, and adapt programme delivery to reach children both within and beyond priority areas.

### Capturing the absolute burden

Effective prioritization depends on robust data systems that identify where the greatest numbers of children affected by wasting are. Population-based surveys such as the DHS and Multiple Indicator Cluster Surveys (MICS) are the gold standard for estimating the prevalence of wasting while also providing essential context on maternal health, socioeconomic conditions and other determinants of child health. The international community and country governments should prioritize maintaining these surveys wherever possible, through a renewed commitment to the DHS and MICS programmes, increased domestic investment or shared financing.

Where sustaining full household surveys is not feasible, countries could use the established operational platforms of DHS and MICS to conduct nutrition-focused surveys. These targeted surveys would use fewer resources and could be conducted more frequently, allowing data collection to better respond to programmatic needs. Furthermore, by focusing specifically on wasting, these surveys should incorporate measurement of MUAC and screening for bilateral pitting oedema alongside (or instead of) WHZ, which would reduce underestimation of the burden of wasting and shift the focus towards the children at the highest risk of death. Alternatively, governments could use World Health Organization (WHO) support, collecting data through the World Health Survey Plus and managing it through the world health data hub.^27^

Routine health data systems are also an underused but vital source of information. Strengthening the ability to capture childhood wasting in electronic medical records and national health information systems, such as the District Health Information System (DHIS2),^28^ could improve the estimation of subnational burdens of wasting over time. One strategy is the adoption of the United Nations Children’s Fund (UNICEF) and WHO standard nutrition package into existing DHIS2 platforms.^28^^,^^29^ This package standardizes screening data across countries and helps local health systems align with global nutrition goals. However, the reliability of data from health information systems is constrained by staff shortages, limited internet connectivity and the inefficiencies of maintaining parallel paper and digital records.^30^^,^^31^ These challenges are compounded by socioeconomic barriers to health-care access and inadequate screening at health facilities. Consequently, current data within health information systems likely underestimate the national burden of wasting and introduce bias across subnational regions. Governments should therefore invest in training health workers and streamline reporting to ensure these data are both accurate and accessible.

Climate and food security monitoring systems, such as the Famine Early Warning Systems Network^32^ and regional meteorological agencies, offer complementary insights for wasting prevention by tracking short-term environmental and livelihood shocks that population surveys cannot. The risk of wasting is characterized by extreme within-year variability,^33^ and reliance on surveys that provide a single annual snapshot can obscure both the true wasting burden and its seasonal drivers.^34^ As a result, multiple within-year observations are essential to identify periods of heightened risk and inform timely, preventive action. However, these data sources also share the limitations of prevalence-based strategies, as many children with wasting live in non-arid urban areas where malnutrition is less directly linked to local climate. Improved data systems should support flexible prioritization that is revisited regularly, particularly in contexts of climate shocks, displacement or funding volatility. Given the inherent limitations of data sources, integrating population-based surveys, health-care use and food security data may yield more accurate geospatial models for policy planning.

### Optimizing prioritization

Ensuring wasting services reach the most vulnerable children requires regular reassessment of geographic targeting strategies. Health ministries should assess whether current priority areas defined in the global action plan accurately reflect where children with wasting live. This evaluation is timely as 2026 marks the end of a 3-year transition phase for the *Global action plan on child wasting*.^35^ Through the Joint Action to Stop Wasting, countries such as Burkina Faso, Ethiopia and Kenya have incorporated new implementation models to accelerate their progress towards global nutrition targets, underscoring the need to assess whether existing prioritization models have driven substantial improvements and are still relevant.

Our case studies show that, while global action plan prioritization has successfully targeted areas of high wasting prevalence, it fails to capture a substantial share of the national burden concentrated in more densely populated non-priority areas. Because non-priority areas have historically received little or no comparable investment, we do not know how much wasting could be reduced in non-priority settings if similar interventions were implemented there. Observed declines in priority areas therefore cannot be interpreted as evidence that prevalence-based targeting is superior to burden-based approaches. Together, these findings demonstrate that prevalence alone is an insufficient basis for geographic prioritization.

Shifting to prioritization based on burden does not mean abandoning investment in high-prevalence areas, which have consistently experienced large reductions in wasting with interventions. Rather, such a move highlights the need to complement these efforts with strategies that account for where the largest numbers of affected children live, including densely populated non-priority settings. We therefore reinforce the case for whole-country nutrition strategies that extend support beyond historically prioritized areas and align resources with the full national burden of wasting. 

### Reaching the most children

Optimizing geographic priorities must be accompanied by system-wide programmatic adaptations that make comprehensive coverage feasible under resource constraints. An approach based on the burden of wasting requires wasting treatment to be strengthened across the entire health system. This approach does not mean simply replicating existing models in new locations, but tailoring strategies based on the specific opportunities and constraints of each context.

Health ministries should conduct programmatic evaluations in all settings, both within established priority areas and in regions with high case numbers that have received limited investment. These assessments should identify gaps in service delivery, missed opportunities for case detection and access barriers to inform context-specific strategies for improving outcomes. In priority areas, evaluations can show how to sustain gains while improving efficiency; in non-priority settings, such evaluations can uncover unused potential within existing infrastructure.

Expanding services that address wasting does not require creating parallel systems. In many settings, meaningful gains could be achieved by strengthening the detection and management of wasting within existing paediatric, maternal, inpatient and outpatient services where high-risk children are frequently seen but not routinely screened or treated.^36^ Urban areas may be ideal for scaling up cost-effective interventions within the Integrated Management of Acute Malnutrition due to robust health infrastructure, staff availability and lower transportation costs for nutrition and health goods. In these contexts, the same level of investment may reach and treat more children than in harder-to-reach settings. Meanwhile, priority areas with a declining prevalence of wasting may benefit from transitioning towards simplified delivery models that maintain coverage while freeing resources for use elsewhere.

Several simplified approaches within the Integrated Management of Acute Malnutrition offer pathways to improve both efficiency and reach without compromising quality of care. MUAC screening by caregivers empowers families to monitor their children’s nutritional status at home,^37^ which allows community health workers to focus on diagnosis verification, training, case management and referrals. While programmes based on MUAC screening only streamline case detection, they can miss children with discordant MUAC and WHZ measurements.^38^ MUAC-for-age protocols may address this issue,^39^ but are difficult to implement in practice. Optimizing the dosage of ready-to-use therapeutic foods can also reduce programme costs without compromising effectiveness. For example, the OptiMA trial in Burkina Faso found that a simplified protocol based on MUAC measurement with gradually reduced dosing of ready-to-use therapeutic foods for both moderate and severe cases of wasting resulted in better recovery than the accepted standard while still lowering overall consumption of ready-to-use therapeutic foods.^40^ Additionally, encouraging local production of ready-to-use therapeutic foods and developing more affordable therapeutic foods could reduce costs while building sustainable supply chains.^41^^,^^42^ Finally, the provision of small-quantity lipid-based nutrient supplements as a preventive measure against wasting has been recommended in settings with high nutritional vulnerability or severe acute food insecurity, including humanitarian emergencies;^43^ however, their high cost-per-case averted may limit widespread use.^44^

## Conclusion

As the nutrition funding landscape changes, existing nutrition programmes and policies must be re-evaluated, given the lasting impact of wasting on survival, development and human capital. Prevalence-based targeting under the global action plan has successfully delivered nutritional services to millions of children with wasting, demonstrating the value of focused investment. However, many countries will not achieve their nutritional targets without tackling the substantial burden of wasting that remains beyond prioritized areas. Such action does not require abandoning effective investments in high-prevalence settings, but rather adopting a flexible, regularly updated prioritization that balances prevalence with absolute burden. Under increasing resource constraints, countries can optimize impact by using context-specific opportunities. By scaling up cost-effective services in densely populated areas with existing infrastructure, while sustaining gains in priority areas through simplified approaches, countries can move towards comprehensive coverage that aligns resources with the full national burden of wasting. This approach can ensure that no child is left behind, regardless of where they live. Lastly, improving wasting management requires reliable, timely data, making sustained investment in population-based survey systems essential for guiding prioritization and tracking progress.
